# Prognostic value of *KRAS* mutation in advanced non-small-cell lung cancer treated with immune checkpoint inhibitors: A meta-analysis and review

**DOI:** 10.18632/oncotarget.17594

**Published:** 2017-05-03

**Authors:** Jung Han Kim, Hyeong Su Kim, Bum Jun Kim

**Affiliations:** ^1^ Division of Hemato-Oncology, Department of Internal Medicine, Kangnam Sacred-Heart Hospital, Hallym University Medical Center, Hallym University College of Medicine, Seoul, Republic of Korea

**Keywords:** non-small-cell lung cancer, immune checkpoint inhibitor, *KRAS* mutation, meta-analysis

## Abstract

Immune checkpoint inhibitors (ICIs) have emerged as a promising treatment option in the fight against advanced non-small-cell lung cancer (NSCLC). *KRAS* is the most frequently mutated oncogene in NSCLC. We performed this meta-analysis to investigate if *KRAS* mutation status affects survival benefits of ICIs in patients with advanced NSCLC. Electronic databases were searched for eligible studies. We included randomized trials with the data of overall survival stratified by *KRAS* mutation status. From 3 eligible studies, 138 patients with *KRAS* mutant NSCLC and 371 with *KRAS* wild-type tumor were included in the meta-analysis. Compared to chemotherapy with docetaxel, ICIs improved overall survival in patients with previously treated *KRAS* mutant NSCLC (hazard ratio = 0.64 [95% confidence interval, 0.43–0.96], *P* = 0.03). For patients with *KRAS* wild-type NSCLC, however, ICIs did not prolong overall survival over that with chemotherapy (hazard ratio = 0.88 [95% confidence interval, 0.68–1.13], *P* = 0.30). In conclusion, ICIs as a salvage therapy improved overall survival over that with docetaxel in advanced NSCLC patients with *KRAS* mutation, but not in those with *KRAS* wild-type tumor. These results suggest that *KRAS* mutation status may be a potential biomarker for survival benefits to ICIs.

## INTRODUCTION

Treatment of advanced non-small-cell lung cancer (NSCLC) progressed dramatically with the introduction of targeted agents in the last 15 years. However, lung cancer still remains the leading cause of cancer-related death all over the world [1, 2]. Recently immune checkpoint inhibitors (ICIs) have emerged as a promising treatment option in the fight against advanced NSCLC [3]. The programmed death-ligand 1 (PD-L1) is an immune checkpoint protein expressed on tumor cells or tumor-infiltrating immune cells. The binding of PD-L1 with programmed death 1 (PD-1) receptors on activated T-cells induces tumor immune escape by downregulating anti-tumoral T-cell function [4, 5]. Thus, inhibition of the PD-1/PD-L1 pathway can induce immune response to cancer by restoring the T-cell activity [6]. ICIs refer to the anti-PD-1/PD-L1 antibodies which were engineered to block PD-1/PD-L1-mediated inhibitory signals. A number of clinical trials in advanced NSCLC have shown that ICIs could derive superior survival outcomes, compared to standard chemotherapy [7–12].

In general, patients with PD-L1 expression on tumor cells and/or tumor-infiltrating immune cells showed better outcomes, compared with those with no PD-L1 expression [7–10]. Because patients with no PD-L1 expression can benefit from ICIs [11], however, PD-L1 expression is not perfect predictive biomarker. Thus, the complexity of tumor-immune interactions requires other biomarkers in addition to or beyond PD-L1.

Tumor mutational burden has been proposed as a potential marker for response to ICIs in advanced NSCLC [13, 14]. High mutational load may be associated with the increase of neo-antigens recognized by T cells to mount antitumor T-cell responses [15]. Thus, high mutational burden contributes to tumor immunogenicity and may affect response to ICIs [6]. *KRAS* is the most frequently mutated oncogene in NSCLC. Lung cancers harboring *KRAS* mutations show prominently increased mutation burden [16]. Subgroup analysis of the CheckMate 057 trial showed that patients with *KRAS* mutation were more like to benefit from nivolumab in term of an improved overall survival (OS) [9]. In other studies with ICIs [10, 11, 17], however, *KRAS* mutational status was not significantly associated with survival benefit of ICIs.

Therefore, it is unclear whether the efficacy of ICIs in patients with advanced NSCLC is associated with *KRAS* mutation. We performed this meta-analysis to investigate if *KRAS* mutation status affects the survival benefits of ICIs in patients with advanced NSCLC.

## RESULTS

### Results of search

**Figure 1:**
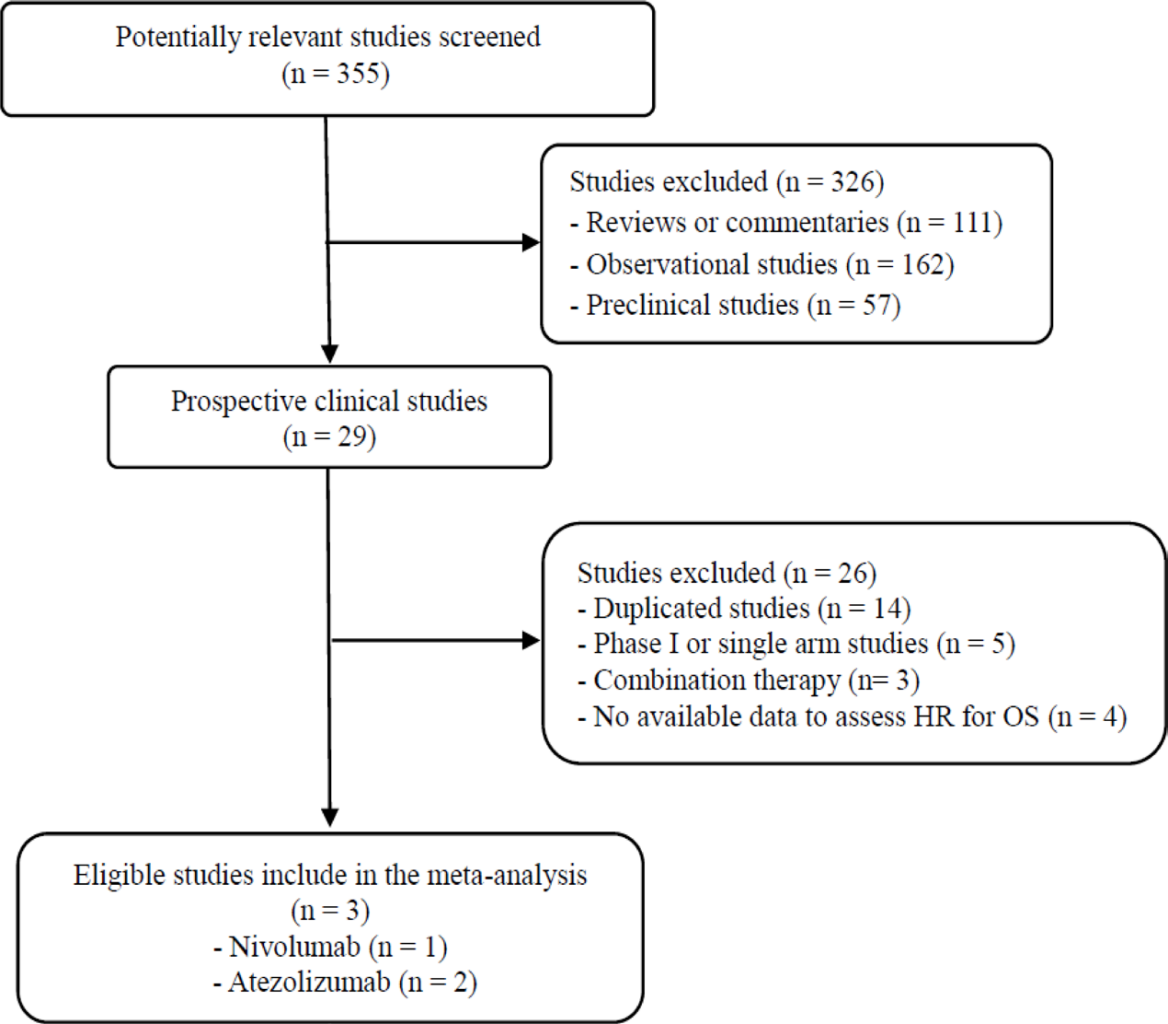
Flowchart of search process

Figure 1 shows the flowchart of studies through the selection process. A total of 355 studies were screened according to the searching strategy; 326 were excluded after screening the titles and abstracts. Out of the remaining 29 potentially relevant prospective studies, 23 were excluded according to the inclusion criteria: four trials had no data to assess hazard ratio (HR) or 95% confidence interval (CI) of OS stratified by *KRAS* mutation status [7, 8, 12, 17]. Finally, three randomized phase 2 or 3 studies were included in the meta-analysis [9–11].

### Characteristics of the eligible studies

Table 1 summarizes the relevant characteristics and survival outcomes of the included studies. All the three studies had been conducted in patients with previously treated NSCLC [9–11]. ICIs used in the studies included an anti-PD-1 antibody (nivolumab) and ananti-PD-L1 antibody (atezolizumab). Docetaxel was used for chemotherapy in all the studies. Tests for *KRAS* mutation were performed only in 519 (30.2%) of 1,719 patients enrolled in the three studies. The *KRAS* mutation rate in the tested tumors was 28.5% (148/519).

**Table 1:** Summary of the three eligible studies

Author Study name (year)	Phase	Setting	PD-L1 cut-off	Treatment (Immunotherapy vs. chemotherapy)	KRAS status	No. of patients	HR for OS (95% CI)
Borghaei *et al*. CheckMate 057 (2015)	3	2nd-line	Any	Nivolumab 3 mg/kg q 2weeks vs. docetaxel	MT WT	62 123	0.52 (0.29–0.95) 0.98 (0.66–1.48)
Fehrenbacher *et al*. POPLAR (2016)	2	2nd-or 3rd-line	Any	Atezolizumab 1200 mg q 3weeks vs. docetaxel	MT WT	27 45	0.95 (0.34–2.64) 0.73 (0.33–1.63)
Rittmeyer *et al*. OAK (2016)	3	2nd-or 3rd-line	Any	Atezolizumab 1200 mg q 3weeks vs. docetaxel	MT WT	59 203	0.71 (0.38–1.35) 0.83 (0.58–1.18)

PD-L1, programmed death-ligand 1; HR, hazard ratio; MT, mutant-type; WT, wild-type; OS, overall survival; CI, confidence interval

### Overall survival of immunotherapy versus chemotherapy in the *KRAS* mutant and wild subgroups

**Figure 2:**
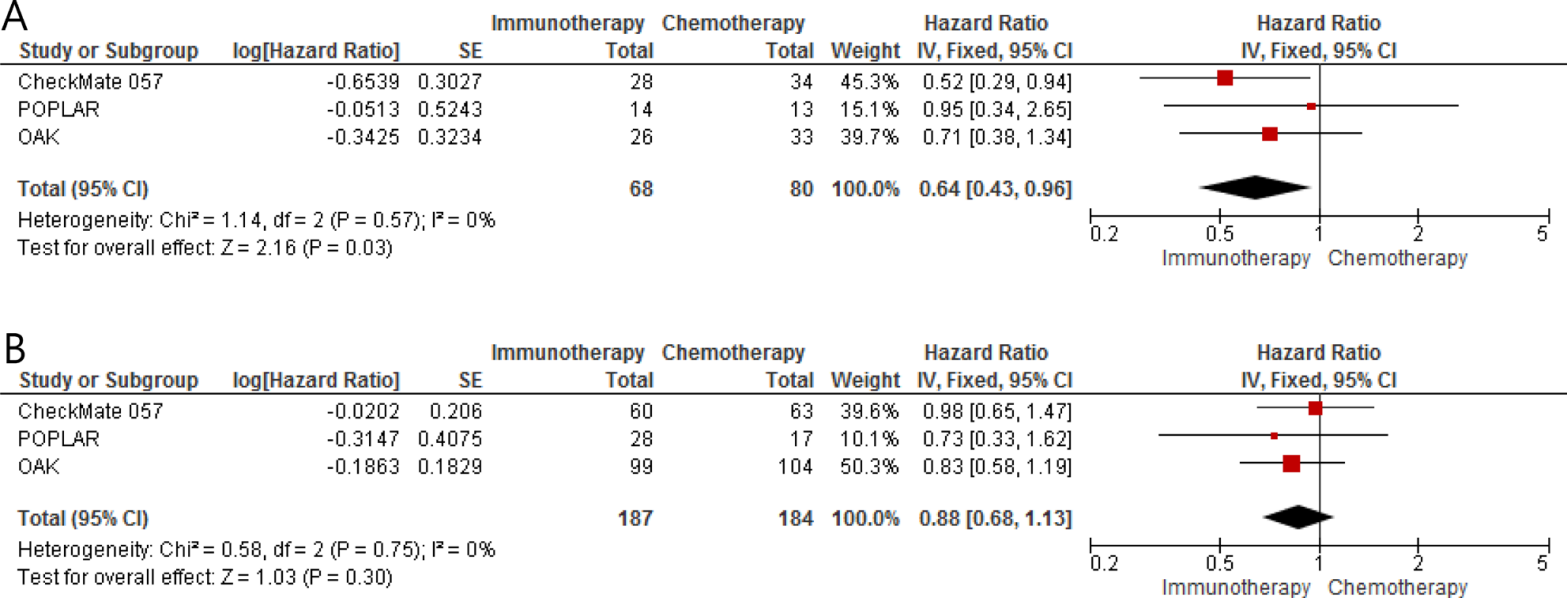
Forest plots of hazard ratios comparing overall survival of immune checkpoint inhibitors versus chemotherapy as salvage therapy in (**A**) patients with *KRAS* mutant NSCLC and (**B**) patients with *KRAS* wild-type tumor.

From the three studies [9–11], 138 patients with *KRAS* mutant NSCLC and 371 with *KRAS* wild-type tumor were included in the meta-analysis of HRs and 95% CIs for OS. Compared to chemotherapy with docetaxel, ICIs improved OS in patients with previously treated *KRAS* mutant NSCLC (HR = 0.64 [95% CI = 0.43–0.96], *P* = 0.03) (Figure 2A). We used the fixed-effect model because there was no significant heterogeneity (*X*^2^ = 1.14, *P* = 0.57, *I^2^* = 0%). For patients with *KRAS* wild-type NSCLC, however, ICIs did not prolong OS over that with chemotherapy (HR = 0.88 [95% CI = 0.68–1.13], *P* = 0.30) (Figure 2B). There was no significant heterogeneity (*X*^2^ = 0.58, *P* = 0.75, *I^2^* = 0%).

## DISCUSSION

In this meta-analysis, we investigated whether survival benefits of ICIs in advanced NSCLC were different according to the *KRAS* mutation status. We found that ICIs as salvage therapy, compared to standard chemotherapy with decetaxel, significantly improved OS in patients with *KRAS* mutant NSCLC, not in those with *KRAS* wild-type tumor.

ICIs have proven survival benefits in advanced NSCLC, but the factors that predict which subtypes of patients will most likely respond to them have not been well established. The PD-L1 expression has been suggested as a predictive marker of clinical efficacy for anti-PD-1/PD-L1 antibodies [7–10, 18]. However, the guidelines and methods to quantify PD-L1 expression are still debated and tumors with no PD-L1 expression can show significant response to ICIs. It is well known that various carcinogens in tobacco smoke are strongly associated with mutagenesis in lung cancer. Thus, lung cancers in tobacco users show a higher mutational burden than those developing in never-smokers [14]. Some studies of ICIs in advanced NSCLC revealed better clinical outcomes among former or current smokers than among non-smokers [8–11]. These observations suggest that mutational heterogeneity of NSCLC may be the key for the success of immunotherapy with ICIs. A recent whole exome sequencing study observed a significant correlation between tumor mutational burden and clinical benefits of pembrolizumab in NSCLC [13]. Considering the findings that cancer types with a relatively high mutational burden showed better outcomes to ICIs [13, 14], specific mutational profile of a given tumor might underlie its potential to respond to ICIs [19].

Recently, a meta-analysis by Lee *et al*. reported that *EGFR* mutation was a potential predictive biomarker for survival benefit to ICIs in advanced NSCLC [20]. In this study, there was a 34% reduction of the risk for death in the *EGFR* wild-type subgroup, but patients with *EGFR* mutant NSCLC showed no survival advantage to ICIs, compared to chemotherapy with docetaxel. These findings may be explained, at least in part, by the result of a recent study with lung adenocarcinoma by Ji *et al*. in which patients with lower PD-L1 expression showed significantly higher *EGFR* mutation rate [21]. On the other side, *EGFR*-mutated NSCLC might have low mutation burden compared to *EGFR* wild-type NSCLC.

Whether NSCLC with *KRAS* mutation have different immunogenicity and hence result in different tumor responses to ICIs is another vital question. Recently Dong *et al*. reported that lung adenocarcinoma with *KRAS* mutations had increased mutation burden [16]. Thus, we assumed that patients with advanced NSCLC harboring *KRAS* mutations might show better outcomes to ICIs than those with *KRAS* wild-type tumor. In this meta-analysis, ICIs as second- or third-line therapy prolonged OS over that with docetaxel in patients with *KRAS* mutant NSCLC. In patients with *KRAS* wild-type NSCLC, however, there was no significant survival difference between immunotherapy with ICIs and chemotherapy with docetaxel. These results suggest that *KRAS* mutation status may be a potential biomarker for clinical benefits to ICIs in advanced NSCLC.

Our study has several limitations. First, this meta-analysis included a small number of studies conducted in more than second-line treatment setting. Moreover, *KRAS* mutation tests were performed only in a small portion of the enrolled patients. Second, the current study included heterogeneous patients with various levels of PD-L1 expression. *KRAS* mutation status might affect survival outcomes of ICIs differently according to the PD-L1expression level. Lastly, this meta-analysis could not include two randomized phase 3 studies that had tested ICIs as first-line treatment for advanced NSCLC because no relevant data on *KRAS* mutation were available [12, 17].

In conclusion, this meta-analysis found that ICIs as a salvage therapy improved OS over that with docetaxel in advanced NSCLC patients with *KRAS* mutation, but not in those with *KRAS* wild-type tumor. These results suggest that *KRAS* mutation status may be a potential biomarker for survival benefits to ICIs. Considering this meta-analysis was based on a limited amount of data, however, further studies are still warranted to evaluate the impact of *KRAS* mutation on the efficacy of ICIs in patients with advanced NSCLC. We also expect that recent advances in the next-generation sequencing will allow for the identification of more accurate biomarkers for clinical benefits to immunotherapy with ICIs.

## MATERIALS AND METHODS

### Searching strategy

The following terms were adopted for literature searching: ‘immune checkpoint inhibitor or immunotherapy’, ‘nivolumab or pembrolizumab or atezolizumab or ipilimumab’, ‘advanced or metastatic’, ‘non-small-cell lung cancer or NSCLC’, and ‘PD-1 or PD-L1.’ We carried out a systematic search of electronic databases, such as PubMed, MEDLINE, EMBASE, and Google Scholar. In addition, we manually searched abstracts presented in the ESMO 2016 Congress or IASLC 17^th^ WCLC. We also looked into all the references of identified relevant articles and reviews. When the data were unclear or incomplete, we tried to contact the corresponding authors.

Eligible studies needed to meet the inclusion criteria: randomized controlled trials in advanced NSCLC; randomization of patients to either immunotherapy with ICI or chemotherapy; performing subgroup comparison of PFS or OS by *KRAS* mutation status; providing HR and its 95% CI.

### Data extraction

The following data were collected from each eligible study: first author's name, year of publication, study phase, number of patients, treatment setting and regimen, PD-L1 expression level, PFS or OS stratified by *KRAS* mutation status and their HRs with 95% CIs. Data extractions were carried out independently by two authors (BJK and HSK). If the two authors could not reach a consensus, the other (JHK) was consulted to resolve the discrepancies.

### Statistical analyses

Statistical values used in the meta-analysis were obtained directly from the original articles or abstracts. HRs with 95% CIs for OS were pooled. Heterogeneity across studies was assessed using the *I*^2^ inconsistency test and chi-square-based Cochran's *Q* statistic test in which *P* < 0.1 indicates the presence of significant heterogeneity. The fixed-effect model (Mantel-Haenszel method) was used to calculate the pooled HR when substantial heterogeneity was not observed. In cases of potential heterogeneity, the random-effects model (DerSimonian-Laird method) was adopted. The final result was reported with HR with 95% CI. All *P*-values were two-sided and *P* < 0.05 was considered statistically significant. RevMan version 5.2 software was used to report outcomes.
